# A Qualitative Inquiry into the Human Library Approach: Facilitating Social Inclusion and Promoting Recovery

**DOI:** 10.3390/ijerph17093029

**Published:** 2020-04-27

**Authors:** Chi Kin Kwan

**Affiliations:** Department of Social and Behavioural Sciences, City University of Hong Kong, Tat Chee Avenue, Kowloon Tong, Hong Kong; ckkwan@cityu.edu.hk

**Keywords:** social inclusion, mental health, anti-stereotyping, human library

## Abstract

The key to the successful social inclusion of people recovering from mental illness is mutual understanding with other community members. To promote such social inclusion, the human library approach has been adopted by a group of practitioners based in Hong Kong. Through a review of this community mental health initiative, this study explores the relevance and usefulness of this approach in a mental health setting. A collaborative inquiry-based research method was adopted to explore the human library approach in practice. A practitioner inquiry group was conducted with four social workers and three peer support workers to examine their experience of running the human library. Thematic analysis and member checks were used to identify important themes. The practitioners’ reports of their experiences showed that the human library is well suited to facilitating social inclusion and promoting mental health recovery. Community members and people in recovery can benefit from participating in a human library, and the two sides can become connected through mutual understanding. However, possible risks for people in recovery were also identified. This study argues that the human library deserves consideration as an approach to facilitating social inclusion and promoting recovery. Its effectiveness and benefits are evident, especially compared with large-scale one-way intervention approaches. A clinical practice manual should be developed to inform future practitioners of the value of the human library approach in mental health settings.

## 1. Introduction

The human library approach was developed as a community intervention strategy to facilitate the optimal social inclusion of people in recovery from mental illness (“in recovery”) [1]. The first human library was set up in 2000 in Denmark with the theme “Stop the Violence,” and the approach has continuously evolved since [2]. According to Johannsen, the human library is “an innovative method designed to promote dialogue, reduce prejudices, and encourage understanding” [1]. By participating in the human library, readers (visitors) can engage in a dialogue with “living books,” or storytellers, whom they may seldom meet in daily life [3]. Following this rationale, the human library approach has been promoted in other countries and adopted in various fields [3,4].

The human library has pragmatic relevance to the field of mental health services. People in recovery encounter difficulties not only due to their symptoms but also from other people’s stereotypes and prejudices about mental illness [5,6]. Stigma and misunderstanding of mental illness remain serious issues [7,8,9,10], as they are often considered barriers to social inclusion [11,12]. Stigma may even discourage people in recovery from seeking professional support [13]. A lack of personal contact between people in recovery and the public is one of the reasons for stigma towards people with mental health issues [14], and, therefore, facilitating intergroup contact between people in recovery and other community members may be helpful. This idea is consistent with the intergroup contact hypothesis formalized by Allport [15], who suggested that positive contact between groups can reduce negative prejudice. Moreover, recent studies have shown that face-to-face contact can be effective in reducing mental health stigma [16]. As the human library approach focuses on facilitating intergroup contact [17], it seems to be a promising intervention method of tackling the stigma of mental health. Given that impersonal large-scale mental health care promotions targeting the stigma of mental illness may not be very effective [18], exploring the application of the human library as an alternative approach may be valuable.

In human library practice, the word “dialogue” is often emphasized because of the rationale that personal contact is powerful and can challenge negative stereotypes [19]. The dialogic nature of a human library distinguishes it from large-scale one-way mental health talks delivered mainly by mental health professionals. In this dialogic environment, living books have control of the conversation according to the reader’s interest [20]. Unlike health care talks, in which the content is usually prepared, the human library allows spontaneous interactions between living books and readers [21]. As such interactive engagement is considered an important element of contact-based mental health intervention [22], it is important to understand whether and how this relationship can be maintained when the human library approach is used to promote greater social equity for people in recovery.

The human library approach has the potential to significantly improve social inclusion. The facilitation of book–reader dialogue may reduce the social distance between people in recovery and people without mental health issues. As reported by Frey and Powell [23], human libraries can help improve social relationships. In their study, the participants gained insights into community relationships, such as, “[p]eople are friendly if you open up” and “I really liked the chance to get to know a member of the Center community a little better” (pp. 74–75). By adopting the human library in a mental health setting, the “us–them” demarcation between people in recovery and other community members may be reduced. 

The human library approach has other potential benefits for both readers and living books. Visitors (readers) to a human library may gain first-hand knowledge by meeting people who have experienced mental health issues (living books). As Schur and her colleagues [24] found, prior contact with people with disabilities can reduce negative stereotypes. An evaluation study of a contact-based anti-stigma intervention program in Canada [25] showed that personal contact with people who have experienced mental health difficulties can help challenge stereotypes in a school setting. Another study conducted in Canada suggested that contact-based strategies work for reducing mental illness-related stigma in pharmacy students [26]. 

Storytelling can be an enabling process for the living books. The human library approach helps living books (who are usually socio-economically disadvantaged group members) to connect with readers (other community members) [27]. According to Dobreski and Huang [27], by participating in dialogue, living books have opportunities to reflect on and review their past experiences. Hence, dialogue can be a learning experience for living books as well as readers [28]. 

Although intergroup contact has benefits, the quality of the interaction matters. Merely bringing two groups together is not enough to overcome stigma and avoid misunderstandings. Without careful planning and arrangement, intergroup contact can “serve to confirm rather than undermine negative attitudes towards outgroups” (p. 201) [29]. To avoid such negative consequences when implementing the human library approach in a mental health setting, a closer look at the approach is vital.

Although the human library approach is becoming popular globally, it has received little exploration in scholarly literature [3,19,26,30]. Although this approach may potentially improve social inclusion, its implementation to reduce the stigma of mental illness is still in the beginning stages. It remains to be seen how human libraries may contribute to the promotion of mental health care. 

Guided by the general aim of the human library approach, a mental health organization in Hong Kong conducted a 1-year trial run of this intervention practice. The author was invited by this organization to conduct a program evaluation of this practice. Initially, the organization was eager to prove the effectiveness of this approach in facilitating social inclusion. However, because the project was still in its beginning stages, it was necessary to start with a deep qualitative inquiry into the approach before conducting large-scale quantitative program evaluation. Against this backdrop, the first-year experience of practice of the human library was used as the case in the current study. As the application of the human library in mental health care is still not very common and such practice experience has not been well documented in the literature, particular attention was given to understanding practitioners’ experience in adopting this intervention practice in a mental health service context. 

## 2. Materials and Methods

My aim was to discover relevant knowledge about the human library approach by reviewing and reflecting on practitioners’ experiences [31]. A practitioner inquiry group was formed because this research method helps practitioners to review their practice [32]. Practitioner inquiry is regarded as a work-based learning process that can generate contextualized knowledge [33]. The inquiry process itself is empowering, as practitioners can discover knowledge by themselves and for themselves [34]. Although the knowledge generated might have limited generalizability, it can lay the foundation for large-scale quantitative studies in the future. 

Ethical approval was obtained from the author’s affiliated university (No. 2-19-201911-01). Two sessions of practitioner inquiry group discussion were held at the meeting room of the service center, a place with which the inquiry group members were familiar. The inquiry group members were invited by the organization. There were seven participants: four social workers and three peer support workers. The inclusion criteria were having served the organization as paid staff and having participated in organizing and offering the human library for at least 6 months. To ensure the reliability of the findings [35], two types of practitioners were included. The four social workers had participated in the human library as librarians, and the three peer support workers, who experienced mental health problems (schizophrenia and depression), had participated as living books. The mean age of the inquiry group members was 40, and their average length of experience in mental health service was 8.3 years.

Informed consent was obtained from the inquiry group members, who were regarded as equal partners rather than passive research subjects. The author, who had several years of experience of working with people in recovery but was not involved in the implementation of the human library, served as the moderator of the inquiry group. An initial discussion was conducted with one of the team members (also a group member) to determine the inquiry group’s arrangement, following the recommendations of Reason [36]. As the teammates were not familiar with the practitioner inquiry method, basic information about this method was provided to them. 

At the beginning of the first session, the inquiry group members discussed the purposes of the group. The agreed-upon goal was to identify the nature and possible usefulness of the human library in promoting mental health. The group hoped to produce a manual for the human library approach that listed its basic principles and procedures to support and improve future practice. The moderator followed a predetermined topic guide to invite group members to critically discuss the nature of the human library and the ways it could be useful based on their experience of running it. Open-ended questions were developed by the author according to the objectives of the study. Sample questions included “What can the human library achieve?” and “What do you think about the usefulness of human library practice?” Points of difference were celebrated, as one aim was to avoid possible problems caused by “group think” [37]. Opportunities were given for the group members to discuss anything they regarded as relevant. The first session lasted for approximately 90 min. The discussions were digitally recorded, and a verbatim transcription of each recording was produced.

Thematic analysis was used [38] to analyze the data. Initial codes were generated after repeated independent readings of the transcripts by the author and a research assistant. Analysis was undertaken by collating all of the codes. Possible themes related to the research objective were also identified. Themes across the seven participants thus emerged, and these themes were reviewed and re-named for meaning before the final article was produced. Negative case analysis [39] was conducted and possible drawbacks of this approach were identified. To enhance the validity of the data, a member check group session was conducted, lasting for approximately 1 h. The author reported the initial findings (i.e., the five major themes) to the whole inquiry group. All of the members agreed on the themes, although some of them offered remarks on the operational details (e.g., the length of each session, the total number of living books available each time). Although these suggestions were not included in this article, they were useful for the production of the manual of human library practice. In the writing process, a representative of the inquiry group was involved as an advisor and provided comments and suggestions. We discussed the data and our ideas before preparing this article. 

## 3. Results

On the whole, the participants held favorable attitudes toward the human library approach, which they considered to be well suited to the mental health service context. The human library approach was perceived to be effective in achieving the service organizer’s initial objective of facilitating social inclusion. The participants identified benefits of being living books that may positively influence people’s recovery from mental illness, and possible negative effects on living books were also noted. Five interrelated main themes were identified:

1. The dialogic nature of the human library generates positive effects.
2. The human library can reduce prejudice against people in recovery.
3. The human library can help remove the barriers between people in recovery and other community members.
4. As living books, people in recovery can obtain insights and emotional support from readers.
5. People in recovery may get hurt in the interaction process.

### 3.1. The Dialogic Nature of the Human Library Generates Positive Effects

Unsurprisingly, the participants highlighted the dialogic nature of the approach. However, they emphasized that the human library approach is much more effective than large-scale one-way intervention approaches. The group members mostly compared the human library approach with conventional educational activities.

“A group format in the human library allows more in-depth [interaction], achieving an effect that cannot be achieved by a conventional health talk. Talks can accommodate a large audience, but the content tends to be general, and it cannot allow the direct, in-depth contacts that the human library can achieve.”

*(David, male, social worker)*

The participants considered the dialogic nature of the human library to be essential to facilitating better interaction, making room for creativity:

“The human library focuses on interaction, and there are few restrictions on the dialogue, allowing more creativity.”

*(Billy, male, peer support worker)*

The group members also stressed that having direct encounters between people in recovery and other community members was important. They suggested that the human library allows people in recovery to represent themselves without an intermediary:

“The human library indeed gives community members one more channel to understand another side of our people in recovery, who often rely on newspapers and whispered rumor. They might not have a chance to engage in an equal dialogue in a safe environment.”

*(Peter, male, social worker)*

The group members compared the human library with the conventional mental health promotion approach driven by mental health professionals:

“For the usual practice of community education, the voices and experiences of people in recovery may not be reflected. But I believe that [people in recovery] participate more [in the human library].”

*(May, female, peer support worker)*

The peer support workers pointed out that the human library is conducted flexibly on an ad hoc basis. Living books can adapt and tailor the sharing content to readers immediately rather than describing a complete narrative:

“In a talk, listeners listen passively. As speakers, we are asked to tell our whole story. But in the human library, it does not matter whether the sharing is complete or not. The important thing is to let readers know what they want to know.”

*(Billy, male, peer support worker)*

Another group member stated that the content that people in recovery prepare for sharing is relatively unimportant:

“The thing that matters most for the human library is people’s interaction.”

*(David, male, social worker)*

### 3.2. The Human Library Can Reduce Prejudice against People in Recovery

All of the group members agreed that the human library can reduce readers’ discrimination against people in recovery, thereby promoting diversity. When asked what the human library can achieve, a peer support worker reported the following:

“We can indeed live very well in the community, inclusively. By showing that, pointless discrimination can hopefully be reduced.”

*(Billy, male, peer support worker)*

Group members stressed that the benefits to readers went beyond an intellectual understanding of people in recovery:

“[The human library] allows a higher level of involvement of people in recovery in the community…that can help eliminate people’s stereotypes. This can also be achieved, not because of telling people this and that about mental health; the important point here is the inclusion.”

*(May, female, peer support worker)*

The first-hand sharing by people in recovery is good evidence that they are part of the community. Some members believed that the human library can reduce intergroup prejudice by publicly portraying accurate and positive images of people in recovery:

“[Readers] gain more understanding of the living books, people in recovery, which is in-depth. They can identify a lot of strengths that people in recovery possess.”

*(Peter, male, social worker)*

Peter further explained the importance of face-to-face personal contact in the human library. He said the human library:

“allows people to understand what people in recovery feel and experience, so that [their] acceptance will be enhanced.”

*(Peter, male, social worker)*

### 3.3. The Human Library Approach Can Help Remove the Barriers between People in Recovery and Other Community Members

In addition to the benefits that readers and living books obtain, the group members believed that interaction in the human library may help address the problem of “othering,” breaking down the us–them demarcation between people in recovery and other community members. When asked about the benefits of the human library approach, nearly all of the group members said that it promotes social inclusion:

“It facilitates better mutual understanding [between readers and living books].”

*(May, female, peer support worker)*

May, who had served as a living book, further explained this benefit:

“I think by running the human library, we can gain a better understanding of each other. They [readers] know more about us [people in recovery]. We can know the opposite side’s perspective, knowing how others perceive people in recovery. Both sides can exchange ideas.”

*(May, female, peer support worker)*

Another group member, a social worker, agreed that the human library can facilitate social inclusion. She suggested that positive changes can occur on both sides:

“I believe [the human library] can facilitate social inclusion, considering that they [people in recovery] are part of society. Furthermore, this is consistent with the recovery model, facilitating a win-win situation [for people in recovery and other community members].”

*(Joe, female, social worker)*

### 3.4. As Living Books, People in Recovery Can Obtain Insights and Emotional Support from Readers

Interaction between living books and readers is not a one-way street. Readers can raise issues for living books to think about, and by doing so can promote living books’ personal growth:

“I mentioned a quarrel I had with my [former] boss, in which I almost got into a fight with him. A reader asked me a question: “Has your boss forgiven you?” This question gave me the opportunity to reflect on whether my boss had forgiven me. That was important; how could I ignore it? I realized that I had made my apology, and he had accepted it.”

*(John, male, peer support worker)*

In addition, the peer support workers reported that they experienced validation in the process:

“I recalled that in a dialogue with university students, one of them was also a mother. When she responded to my sharing, my experience was validated because she told me she had a similar experience. By having such a dialogue, I felt that I got a kind of support.”

*(May, female, peer support worker)*

A social worker also recalled the interaction in the human library that offered emotional support for the living books:

“A living book was so excited to tell his story, and [he] became hyper. But then he suddenly became emotional. Perhaps, when he narrated his story, he felt he was so pitiful: no child, no family, lonely and going to die alone. A man in front of him was in tears, and gave a response. The response allowed the living book to realize that someone supported him, and [he was] not alone. This reset his emotions, and he started to become happy again. Simply put, the feedback was so nice.”

*(Ann, female, social worker)*

### 3.5. People in Recovery May Get Hurt in the Interaction Process

In addition to the positive aspects of the human library, group members were asked to describe any possible negative effects it might have in a mental health setting. A common concern raised by the group members was that the interaction may be risky for people in recovery:

“A person who narrates his/her experience may get hurt if there is no response at all. Merely talking [to readers] is just like [playing] a voice recorder, which can make [living books] feel bad.”

*(David, male, social worker)*

Worse still, the responses that readers give may make living books feel emotionally vulnerable. All three peer support workers suggested that living books need to be aware of their own emotions in the process:

“As a living book, there is a need to be emotionally self-sensitive. When we find that we almost collapse, that we may get hurt, then we need to remind ourselves to shift topics. Because the living book has the right not to answer any questions if we do not want to… Perhaps the challenge of the human library, for me, is that you cannot expect what readers want to ask. For example, some questions they ask may be challenging and may trigger our negative emotions.”

*(Billy, male, peer support worker)*

Billy suggested that living books should receive training to make sure that they are psychologically strong enough. 

## 4. Discussion

To the best of the author’s knowledge, this study is the first to examine the usefulness of the human library approach in a Chinese mental health setting. This approach originated as an anti-violence project, and it has since been adopted to promote the experiences of various disadvantaged groups [1]. The present study indicates that the human library approach has value and potential in mental health settings, as it can effectively facilitate social inclusion and promote recovery. 

According to the seven participants in the inquiry group, the human library approach has obvious benefits in challenging stereotypes about people in recovery. Although large-scale mental health promotion is still common, it may not always be very successful [18]. Some people may be skeptical of the positive descriptions of individuals in recovery provided by government entities or health care professionals. As an alternative approach, a human library may be much more convincing, as it gives participants real and direct encounters with people in recovery. Hence, further application of the human library approach to reduce mental health-related stigma should be considered. By introducing a human library to a mental health setting, people in recovery and other community members can develop meaningful relationships, fostering social inclusion. Based on the results reported in this study, participants in a human library can experience meaningful social inclusion. Indeed, the equal-status interactions between living books and readers breaks down the us–them demarcation between people in recovery and other community members. Ideally, by having more dialogue in the community, the line between the two groups can be further blurred and all members can live equally.

Unlike traditional methods of promoting mental health care, the human library approach gives people in recovery opportunities to exchange ideas and develop relationships with other community members in a spontaneous, informal manner. Often, people in recovery are told what is happening to them. The human library can provide a platform for such people to narrate their experiences. Rather than being represented by health care professionals or someone else, people in recovery can speak for themselves in a human library. In this way, they regain the power to interpret their experiences. These features are the essence of what distinguishes the human library approach from large-scale mental health promotion approaches, which are often driven by professionals.

By telling their stories, people in recovery may reconstruct their experiences. The feedback that readers give may also offer insights and comfort, which are valuable for someone in recovery. Simply put, the dialogic process can generate therapeutic effects for people in recovery. According to those who participated in the inquiry group, the human library approach also seems to echo recovery-oriented practice. Rather than focusing on symptoms or treatment, the human library approach demonstrates resilience through the stories of people in recovery [40]. Living books show readers that despite encountering mental health problems, they can still live with their own personal challenges and take part meaningfully in the community. In addition, this approach appears to be a good strategy for facilitating an environment in which people in recovery can regain power. By having a platform to speak for themselves, they may be able to challenge the dominant narrative about their place in society [41]. However, relatively little scholarly attention has been given to this issue faced by individuals in recovery [27]. To better manage the experiences that people in recovery have as living books in the human library, further investigation and discussion are necessary.

The results of the current study have several implications for mental health promotion policy and practice. To begin with, mental health professionals should consider the human library as one of their approaches. It is worth noting that the success of the human library seems to rest on its dialogic nature. Mental health service managers may be inclined to deviate from it to offer interventions at a lower unit cost. However, when many people attend a human library session, interactive dialogue may not be possible, and the positive impact may be greatly reduced. Considering that many people in recovery tend to be vulnerable [42], there should be safeguards to protect them when they narrate their personal stories. The role of the librarian (the person who facilitates the dialogue) is necessary to ensure that the dialogue takes place in a respectful manner, as the “do no harm” principle [43] should always be upheld. Arrangements such as selection, pre-sharing training, and debriefing of living books are all very important. These points will be included in a practice manual published by the inquiry group. 

As noted above, the human library approach has an advantage over large-scale, impersonal mental health wellness methods. Although it was not directly proposed by the inquiry members here, policy makers should consider allocating resources for impactful promotion strategies. Although further empirical evidence may be required to demonstrate the effectiveness of the human library practice in promoting mental health, it seems to be a promising approach that deserves further attention. 

There are several limitations to the present study. It was based on the perspective of a group of practitioners from only one mental health organization in Hong Kong. As the number of inquiry group members was small, the themes identified in the study should be regarded as hypotheses that require further examination with larger samples. As the inquiry group examined the practice over the course of the last year, selective memory bias might have occurred. Although the author directed the inquiry group members to focus on work-based learning rather than on proving the effectiveness of their interventions, the effect of social desirability may still have influenced the study’s results. Likewise, although reflexivity was stressed throughout the research process, the author’s own bias may still have informed the way in which conclusions were drawn. The engagement of the inquiry group members provided valuable feedback against which the author can compare his assumptions. In addition, as only practitioners’ views were considered in the current study, there is a need to quantitatively and qualitatively explore the perspectives and experiences of readers attending the human library. Particular attention should be paid to the extent to which the human library can facilitate social inclusion among people with mental health issues. Finally, a follow-up qualitative study is needed to investigate the effects of individuals’ experience as living books on their recovery.

## 5. Conclusions

Community-based approaches to mental health care may reduce the social exclusion of people in recovery. However, efforts are required to create a harmonious social environment for both people in recovery and other community members [13]. While interventions can be conducted at various levels, from micro to macro, the human library approach seems to be a promising way of facilitating social inclusion and promoting recovery, which supports the view that the further development of this approach in a mental health setting would be advantageous to people in recovery. Stereotypes about people in recovery exist because many people do not have the opportunity to engage in in-depth dialogue with them. The practitioner inquiry group in this study revealed that a human library can serve as an effective intervention to clarify misunderstandings and facilitate social inclusion. The wall between people in recovery and other community members can thus be broken down. People in recovery may also benefit from serving as living books. Engaging in dialogue with other community members may be simple, but such opportunities are valuable for those in recovery. A dialogue on equal footing can allow them to obtain insights and gain emotional support, which are beneficial for their mental health recovery.
